# Protective Effect of *Ribes nigrum* Extract against Blue Light-Induced Retinal Degeneration In Vitro and In Vivo

**DOI:** 10.3390/antiox11050832

**Published:** 2022-04-25

**Authors:** Chae Young Shin, Mun-Hoe Lee, Hyeong-Min Kim, Hee-Chul Chung, Do-Un Kim, Jin-Hee Lee, Kwang Won Jeong

**Affiliations:** 1 Gachon Research Institute of Pharmaceutical Sciences, College of Pharmacy, Gachon University, 191 Hambakmoero, Yeonsu-gu, Incheon 21936, Korea; codud132@gachon.ac.kr; 2 Health Food Research and Development, NEWTREE Co., Ltd., Seoul 05604, Korea; mhlee@inewtree.com (M.-H.L.); hmkim@inewtree.com (H.-M.K.); hchung@inewtree.com (H.-C.C.); dkim@inewtree.com (D.-U.K.); jhlee@inewtree.com (J.-H.L.)

**Keywords:** N-retinylidene-N-retinylethanolamine (A2E), antioxidant, black currant, blue light, drusen, dry age-related macular degeneration (AMD)

## Abstract

Although blackcurrant has several health benefits, such as antioxidant and anti-inflammatory properties, its effects on the retina remain unclear. In this study, we investigated the efficacy of black currant extract (BCE) in an in vitro and in vivo model of dry age-related macular degeneration (AMD) induced by blue light. Dry macular degeneration is characterized by the abnormal accumulation of lipofuscin (e.g., N-retinylidene-N-retinylethanolamine, A2E) in the retina. Blue light (BL) significantly decreased the viability of A2E-laden human retinal pigment epithelial cells (ARPE-19). However, BCE treatment protected ARPE-19 cells from A2E and BL. A2E, which is oxidized by blue light, generates reactive oxygen species in RPE cells. Treatment with BCE significantly decreased (80.8%) reactive oxygen species levels induced by A2E and BL in a concentration-dependent manner. BCE inhibited A2E accumulation in ARPE-19 cells and significantly downregulated the expression of genes increased by A2E and BL in ARPE-19 cells. In vivo, oral administration of BCE (25–100 mg/kg) ameliorated ocular lesions of BL-induced retinal damage in a mouse model and rescued the thickness of the whole retina, photoreceptor segment layer, outer nuclear layer, and inner nuclear layer. The decrease in the number of nuclei in the outer nuclear layer induced by BL was also rescued by BCE. Additionally, BCE administration rescued (40.0%) the BL-induced reduction in the expression level of superoxide dismutase 1. Taken together, our results suggest that BCE may have preventive and therapeutic effects on dry AMD through its antioxidant activity and inhibition of lipofuscin accumulation in the retina.

## 1. Introduction

Age-related macular degeneration (AMD) is an ophthalmic disease in which the macula of the eye degenerates, resulting in vision loss. It occurs most often in people over 50 years of age. In the early stages, the degree of visual impairment is not severe. However, central vision is gradually lost, which interferes with daily life activities. Although the exact pathogenesis has not been elucidated, some theories have been suggested, such as oxidative stress, mitochondrial dysfunction, complement activation, and the inflammatory response. Additionally, several risk factors (age, smoking, family history, and sunlight) have been reported [1].

AMD is classified into the “dry” type, which is non-exudative, and the “wet” type, which is exudative. Wet AMD is characterized by abnormal blood vessels generated in the choroid that penetrate Bruch’s membrane and proliferate, eventually leaking proteins and blood under the macula, resulting in vision damage. Although wet AMD accounts for approximately 10% of all patients with AMD, the rate of blindness is very high. Various therapies have been developed to block angiogenesis, and currently, direct intravitreal injection of anti-vascular endothelial growth factor (VEGF) antibodies is widely used [2]. Dry AMD is characterized by the formation of drusen and photoreceptor cellular debris between the RPE and the Bruch’s membrane. This obstructs the supply of nutrients and causes RPE atrophy, leading to vision problems. Although the blindness rate is low, 80–90% of all patients with AMD have the dry type. As the etiology is currently unknown, treatment methods have not yet been developed. Therefore, antioxidants, such as lutein and zeaxanthin, are used in response to aging, which is considered the main cause [3].

Black currant (BC, *Ribes nigrum* L.) is rich in anthocyanins, which are naturally occurring antioxidants with delphinidin-3-rutinoside (D3R), delphinidin-3-glucoside (D3G), cyanidin-3-rutinoside (C3R), and cyanidin-3-glucoside (C3G) as the main bioactive compounds [4]. BC and BC anthocyanins possess various functions such as anticancer effects, blood vessel protection, and anti-obesity effects [5,6]. BC has also been used as a dietary supplement to improve eye functions. According to previous studies, intake of BC concentrate in clinical trials reduced the dark adaptation threshold and restored and prevented transient refractive alteration and fatigue symptoms caused by video display terminal work in healthy individuals [7]. In addition, the biological mechanisms by which BC can support the improvement in ocular function are also being studied; for example, C3R accelerates rhodopsin regeneration, and D3R relaxes ciliary smooth muscle through regulation of the NO/cGMP pathway [4,8].

Despite its extensive physiological effects, the effectiveness of blackcurrant extract (BCE) in treating dry AMD has not yet been studied. The characteristic ingredients of BC, such as the antioxidants described above, are considered effective for photooxidation, which led us to investigate the potential of BC as a treatment for dry AMD. The efficacy and antioxidant activity of BCE were confirmed in a phototoxicity model of RPE cells induced by blue light (BL). In addition, the physiological signaling affected by BCE was confirmed. Finally, the efficacy of BCE was evaluated in an in vivo model of retinal degeneration induced by blue light. Our results suggest that the antioxidant activity of BCE and the inhibitory activity of lipofuscin accumulation in the retina might exert preventive and therapeutic effects on dry AMD.

## 2. Materials and Methods

### 2.1. Materials

Blackcurrant extract (BCE; ACE40™) standardized based on D3R (148 mg/g) and total anthocyanins (391.26 mg/g) were purchased from JUST The Berries PD Corporation (Los Angeles, CA, USA). The detailed composition of the anthocyanins in BCE is presented in Supplementary Table S1. Lutein was purchased from Acros Organics (Los Angeles, CA, USA). The total anthocyanin content was analyzed using the Health Functional Food Code Test Method 5-59-1, announced by the Ministry of Food and Drug Safety (MFDS) of the Republic of Korea. The content of D3R, D3G, C3R, and C3G in BCE was determined by high-performance liquid chromatography (HPLC)-ultraviolet (UV) using a Waters 2695 HPLC system (Waters, Milford, MA, USA). Briefly, 10 mg of BCE was dissolved in 20 mL of 0.1N HCl and filtered using a 0.45 μm syringe filter. A Jupiter^®^ 4 µm Proteo 90 Å (250 × 4.6 × 4.6 mm; Phenomenex, Torrance, CA, USA) was used for sample separation. The mobile phase consisted of 5% formic acid as mobile phase A and acetonitrile as mobile phase B. The gradient of the mobile phase B was 0 min, 5%; 5 min, 5%; 25 min, 15%; 26 min, 80%; 35 min, 80%; 36 min, 5%; and 50 min, 5%. Other parameters included a column temperature of 35 °C, an injection volume of 10 μL, a flow rate of 1.0 mL/min, and a detection wavelength of 525 nm UV.

### 2.2. Cell Culture

The human retinal pigment epithelial cell line (ARPE-19) was purchased from ATCC (Manassas, VA, USA) and cultured in DMEM/F-12 (Welgene, Daegu, Korea) supplemented with 10% fetal bovine serum (Gibco Island, NY, USA), 100 U/mL penicillin, and 100 μg/mL streptomycin (HyClone™, Logan, UT, USA). Cells were incubated at 37 °C and in an atmosphere containing 5% CO_2_. Cells between passages 3 and 10 were used for all experiments.

### 2.3. Cell Viability Assay

EZ-Cytox (DoGen Bio, Seoul, Korea) was used to assess cell viability based on a previously published method [9]. Briefly, ARPE-19 cells were seeded at a density of 2 × 10^4^ cells/well in 24-well plates. Cells treated with BCE (20–80 μg/mL) for 72 h were washed once with 1 × PBS and incubated at 37 °C in serum-free medium containing EZ-Cytox for 3 h. Absorbance was measured at 450 nm using a microplate reader (BioTek, Winooski, VT, USA). For the BL-induced RPE damage model, cells were monitored using the IncuCyte Zoom imaging system (Essen Bioscience, Ann Arbor, MI, USA) for 24 h after BL exposure, and cell viability was calculated as confluence.

### 2.4. BL-Induced RPE Damage Model in RPE Cells

The in vitro BL-induced RPE damage model was established following a previously published method [9]. Briefly, ARPE-19 cells were seeded at a density of 2 × 10^4^ cells/well in 6-well plates. Cells were treated with N-retinylidene-N-retinylethanolamine (A2E) (5 μM) three times at 48 h intervals. Then, 24 h after the last treatment with A2E, BCE (10–50 μg/mL) was administered twice at 24 h intervals. As previously reported, lutein (15 μg/mL) was used as a positive control [10,11,12]. After 30 min of BL irradiation, the cells were allowed to recover for 24 h.

### 2.5. Measurement of Reactive Oxygen Species (ROS) Production

2′,7′-Dichlorofluorescin diacetate (DCF-DA; Sigma-Aldrich, St. Louis, MO, USA) was used to evaluate the ROS levels in ARPE-19 cells. Briefly, at 24 h after BL exposure, the cells were treated with 10 μM DCF-DA at 37 °C for 10 min in the dark. After washing the cells twice with 1 × PBS, they were visualized using a JuLI™ smart fluorescent cell analyzer (NanoEntek, Seoul, Korea) and quantified using the Image J software v1.53k (National Institutes of Health, Bethesda, MD, USA) [13].

### 2.6. Reverse-Transcriptase Quantitative Polymerase Chain Reaction (RT-qPCR)

At 24 h after BL exposure, total RNA was isolated using the TRIzol^®^ reagent (Invitrogen, Carlsbad, CA, USA) and reverse-transcribed using an iScript cDNA synthesis kit (Bio-Rad Laboratories, Hercules, CA, USA). RT-qPCR was performed on a Roche LightCycler^®^ 480II system using SYBR Green I Master (TOYOBO, Osaka, Japan) with the primers listed in Supplementary Table S2. The mRNA expression levels were normalized to *18S* rRNA levels [14].

### 2.7. A2E Accumulation Assay

A2E accumulation was measured using fluorescence-labeled A2E (A2E-BDP) [15]. Cells seeded at a density of 5 × 10^3^ cells/well in a 96-well white plate were pretreated with BCE (10–50 μg/mL) or lutein (15 μg/mL) for 24 h. Then, cells were treated with A2E-BDP (10 μM) for 24 h. Intracellular A2E levels were measured using a fluorescence microplate reader (VICTOR™ X3; PerkinElmer Inc., Waltham, MA, USA) at 485 nm (excitation) and 535 nm (emission).

### 2.8. Ophthalmological Examination

The day before euthanasia, the pupils were dilated with a mydriatic agent and ophthalmological examination of both eyes was performed using a fundus camera (Genesis-D; Kowa Co. Ltd., Nagoya, Japan). The presence or absence of lesions of retinal pigmentation, retinal hyperemia, and drusen was evaluated qualitatively, and the number of animals with lesions was compared between groups [16].

### 2.9. BL-Induced Retinal Damage Model in Mice

All animal experiments were approved by the Institutional Animal Care and Use Committee of Korea Conformity Laboratories (Approval No. IA21-01649). Sixty BALB/c mice (5 weeks; male) were purchased from Orientbio (Seongnam-si, Korea). All mice were housed at 23–25 °C, 55% ± 15% relative humidity, 12 h light–dark cycle (8 am–8 pm) and given free access to food and water during the experiments. After 1-week of acclimation, mice were randomly divided into six groups (*n* = 10/group) and treated with vehicle, BCE (25, 50, and 100 mg/kg/day), or lutein (4.6 mg/kg/day) for 1 week. After pretreatment, BL-induced retinal damage was induced by irradiating BL (10,000 lux) with vehicle, BCE, or lutein for 2 weeks [9]. The test compounds were administered 30 min prior to BL exposure. Dark room conditions were maintained for 24 h during the BL irradiation period, and BL was irradiated once a day for 1 h. After the BL irradiation period, the test compound was administered for 2 weeks. All mice were euthanized using CO_2_ gas and their eyes were removed and fixed in Davidson’s solution [17]. The groups were as follows: normal group: animals administered vehicle; BL group: animals exposed to BL and administered vehicle; and BL + test compound group: animals exposed to BL and administered BCE (25, 50, and 100 mg/kg/day) or lutein (4.6 mg/kg/day).

### 2.10. Histological Analyses

Both eyes of all mice were fixed in Davidson’s solution for 2 days and then embedded in paraffin. Paraffin-embedded tissues were sectioned at a thickness of 3 μm. H&E staining was performed according to a standard protocol. Sections between 100 and 200 μm from the optic nerve of both eyes were observed at 400× magnification using an Axio Scope optical microscope (ZEISS, Oberkochen, Germany). The thicknesses of the whole retina, photoreceptor segment layer (PSL), inner nuclear layer (INL), and outer nuclear layer (ONL) were measured using the Axio Vision SE64 (ZEISS) program. The number of nuclei in the ONL was analyzed using the Image J software [17]. For immunohistochemistry (IHC), sectioned tissue was stained with an anti-Superoxide dismutase 1 (SOD1) antibody (ab51254, Abcam, Cambridge, UK). Images were acquired using a confocal laser scanning microscope (Nikon, Minato-ku, Japan) and quantified using the Image J software [18].

### 2.11. Statistical Analyses

For the in vitro study, statistical analyses were performed using GraphPad Prism 8 (GraphPad Software, San Diego, CA, USA). Data are presented as the mean ± standard deviation, and significant differences among groups were calculated using one-way analysis of variance (ANOVA). Statistical significance was set at *p* < 0.05. For the in vivo study, statistical analyses were performed using the SPSS 12.0 K program (SPSS, Chicago, IL, USA). Significant differences among groups were analyzed using one-way ANOVA followed by Duncan’s test for homogeneity of variance or Dunnett’s T3 test for heterogeneity of variance. Statistical significance was set at *p* < 0.05.

## 3. Results

### 3.1. Protective Effect of BCE against In Vitro RPE Damage Model

**Figure 1:**
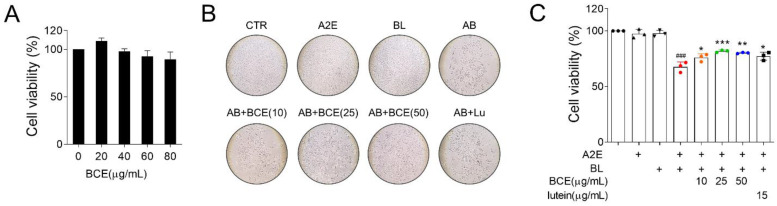
Protective effect of BCE against in vitro RPE damage model. (**A**) Effect of BCE on ARPE-19 cell viability. Cells were treated with BCE (20–80 μg/mL) for 72 h. * *p* < 0.05 vs. CTR. (**B**,**C**) ARPE-19 cells were treated with A2E three times at 48 h intervals, and 24 h after the last treatment, BCE (10–50 μg/mL) was treated twice at 24 h intervals. After 30 min of BL irradiation, the cells were recovered for 24 h. Cells were monitored using the IncuCyte Zoom imaging system. Lutein (15 μg/mL) was used as a positive control. The results are presented as the mean ± standard deviation of three independent experiments (*n* = 3). ^###^ *p* < 0.001 vs. CTR, * *p* < 0.05, ** *p* < 0.01, *** *p* < 0.001 vs. A2E + BL. CTR, no treatment control; A, A2E; BL, blue light; AB, A2E + BL; Lu, lutein (15 μg/mL); BCE (10), BCE, 10 μg/mL; BCE (25), BCE, 25 μg/mL; BCE (50), BCE, 50 μg/mL; A2E, *N*-retinylidene-*N*-retinylethanolamine; BL, blue light; BCE, Blackcurrant extract.

First, we investigated the protective effects of BCE in an in vitro RPE damage (A2E + BL) model. BCE showed no significant toxicity in ARPE-19 cells up to a concentration of 80 μg/mL and based on this, 50 μg/mL was selected as the highest concentration (Figure 1A). A2E + BL treatment was performed as previously described [9]. Cells exposed to A2E or BL showed no remarkable difference in viability compared with the control group. However, cells exposed to both A2E and BL showed significantly decreased viability compared with the control group. In contrast, BCE and lutein used as a positive control significantly inhibited A2E- and BL-induced cell death (Figure 1B). These results indicate that BCE protects RPE cells from the phototoxicity triggered by A2E and BL.

### 3.2. Antioxidant Effect of BCE against In Vitro RPE Damage Model

**Figure 2:**
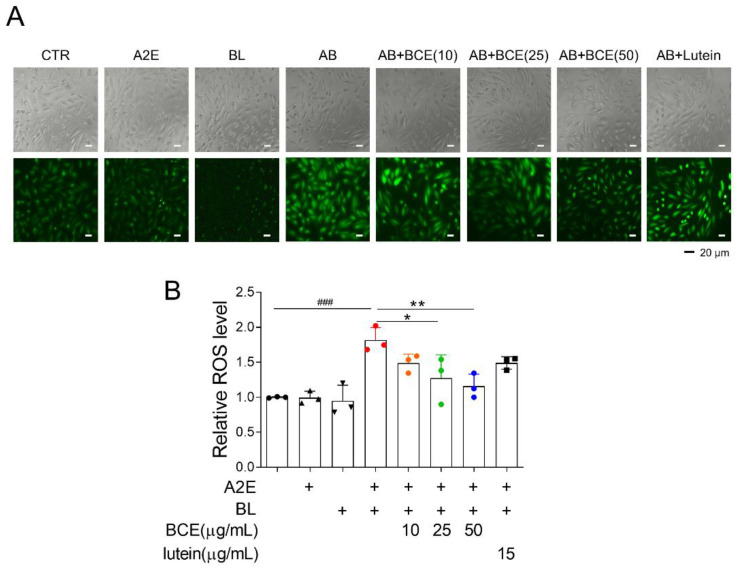
Antioxidant effects of BCE against the in vitro RPE damage model. (**A**,**B**) ARPE-19 cells were treated with A2E three times at 48 h intervals, and 24 h after the last treatment, BCE (10–50 μg/mL) or lutein (15 μg/mL) was treated twice at 24 h intervals. After 30 min of BL irradiation, the cells were recovered for 24 h. Cells were treated with DCF-DA (10 μM) for 10 min in serum-free medium. After washing with 1 × PBS twice, the cells were monitored using JuLI™ smart fluorescent cell analyzer. The relative ROS level was quantified using Image J. The results are presented as the mean ± standard deviation of three independent experiments (*n* = 3). ^###^ *p* < 0.01 vs. normal group, * *p* < 0.05, ** *p* < 0.01 vs. A2E + BL.

Next, we attempted to elucidate the mechanism underlying the retinal cell protective effect of BCE. Dry AMD is associated with oxidative stress [19]. Additionally, it has been reported that in BL- and A2E-induced RPE damage models, ROS are generated by photooxidation of A2E, which activates downstream cell death pathways (e.g., nuclear factor kappa B (NF-κB) pathway, p53 pathway, and apoptosis) [20]. Therefore, we examined whether BCE had an antioxidant effect in a BL-induced RPE damage model. As previously reported, A2E and BL increased ROS levels 1.8 times compared with the control group in ARPE-19 cells. Treatment with BCE significantly decreased ROS levels in a concentration-dependent manner compared with the A2E + BL group. Lutein also decreased ROS level (Figure 2A,B). These results suggest that BCE has an antioxidant effect on RPE cells in the A2E + BL model, and that this effect acts as one of the mechanisms for inhibiting cell death by photooxidation.

### 3.3. Inhibition of A2E Accumulation by BCE in ARPE-19 Cells

**Figure 3:**
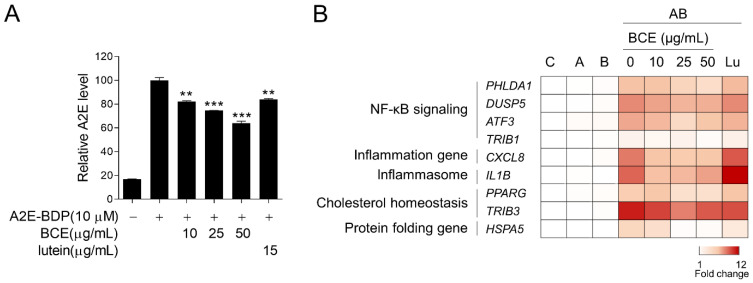
Inhibition of A2E accumulation by BCE in ARPE-19 cells. (**A**) ARPE-19 cells pretreated with BCE (10–50 μg/mL) or lutein (15 μg/mL) were treated with A2E-BDP (10 μM) for 24 h. The relative A2E level was quantified by measuring their fluorescence at 485 nm (excitation) and 535 nm (emission) using a fluorescence microplate reader. The results are presented as the mean ± standard deviation of two independent experiments (*n* = 2). ** *p* < 0.01, *** *p* < 0.001 vs. A2E-BDP. (**B**) Heatmap generated from RT-qPCR results performed on the in vitro RPE damage model. After 24 h of BL exposure, the total RNA was isolated using TRIzol reagent. cDNA synthesized from mRNA was used for RT-qPCR. The mRNA levels were normalized to *18S* rRNA levels. Values represent the log2 fold-change relative to CTR. C, control; A, A2E; B, blue light; AB, A2E + BL. *ATF3*, activating transcription factor 3; *CXCL8*, C-X-C motif chemokine ligand 8; *DUSP5*, dual specificity phosphatase 5; *HSPA5*, heat shock protein family A (Hsp70) member 5; *IL1B*, interleukin 1 beta; *PHLDA1*, pleckstrin homology like domain family A member 1; *PPARG*, peroxisome proliferator activated receptor gamma; *TRIB1*, tribbles pseudokinase 1; *TRIB3*, tribbles pseudokinase 3.

Next, we examined the effect of BCE on A2E accumulation in the RPE cells. ARPE-19 cells were pretreated with BCE for 24 h before treatment with fluorescence-labeled A2E (A2E-BDP). BCE treatment inhibited the accumulation of A2E in ARPE-19 cells in a dose-dependent manner. Lutein also significantly inhibited A2E accumulation (Figure 3A). A2E-laden ARPE-19 cells have been reported to show changes in the expression of several genes when exposed to BL. As BCE inhibited the accumulation of A2E in ARPE-19 cells, it was thought to affect various downstream gene expression pathways induced by BL. We used RT-qPCR to confirm whether BCE had any effect on these genes. The expression of genes related to NF-κB signaling, inflammation, inflammasome, cholesterol homeostasis, and protein folding was greatly increased by A2E and BL. BCE and lutein significantly downregulated the expression of genes induced by A2E and BL in ARPE-19 cells (Figure 3B and Supplementary Figures S1–S4). These results suggest that BCE protects cells from phototoxicity by inhibiting the accumulation of A2E in RPE cells, thereby blocking the activation of various sub-cytophysiological pathways induced by the photooxidation of A2E.

### 3.4. BCE Ameliorates Ocular Lesions of BL-Induced Retinal Damage Mice Model

To examine whether BCE could alleviate the ocular lesions caused by BL irradiation, BCE was orally administered to a BL-induced retinal damage mouse model for 5 weeks. Ophthalmological examination was performed using a fundus camera (Table 1). Mice with lesions of retinal pigmentation, retinal hyperemia, and drusen were not observed in the normal group. In contrast, in the group exposed to BL, there were six and five mice with lesions in the right and left eyes, respectively. In the BCE-treated group, a dose-dependent decrease in the number of subjects with lesions of retinal pigmentation, retinal hyperemia, and drusen was observed. These data suggest that BCE ameliorates ocular lesions caused by BL irradiation.

### 3.5. Protective Effect of BCE on BL-Induced Retinal Degeneration

**Figure 4:**
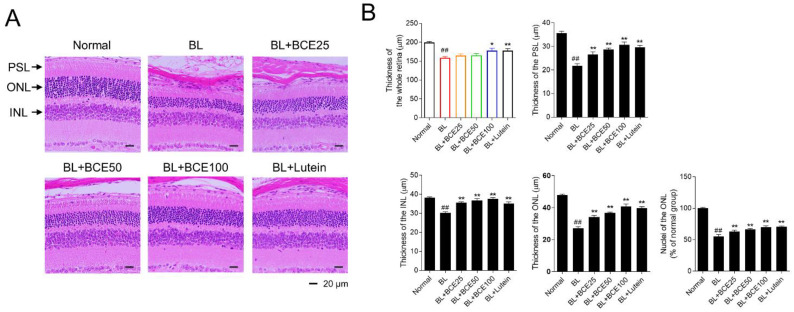
Protective effects of BCE on BL-induced retinal degeneration. (**A**) H&E staining for the evaluation of histological changes. Sections between 100 and 200 μm from the optic nerve of both eyes were observed. (**B**) Measurement of the whole retina, photoreceptor segment layer (PSL), outer nuclear layer (ONL), inner nuclear layer (INL) thickness, and number of nuclei in ONL. The results are presented as the mean ± standard error of the means (*n* = 10). ^##^ *p* < 0.01 vs. normal group, * *p* < 0.05, ** *p* < 0.01 vs. BL group.

To further investigate the protective effect of BCE on retinal degeneration in the BL-induced retinal damage mouse model, retinal degeneration was evaluated by image analysis of tissues stained with H&E (Figure 4A). In the BL-exposed group, the thicknesses of the whole retina, PSL, ONL, and INL were significantly (*p* < 0.01) reduced compared to those in the normal group. Additionally, the number of photoreceptor cell nuclei in the ONL of the BL group was significantly (*p* < 0.01) lower than that of the normal group. However, the administration of BCE (25, 50, and 100 mg/kg/day) in a dose-dependent manner (*p* < 0.05) rescued the thickness of the whole retina, PSL, ONL, and INL (Figure 4B). These results indicated that BCE exerted protective effects against BL-induced retinal degeneration in mice.

### 3.6. Effect of BCE on SOD1 Expression in BL-Induced Retinal Damage Model in Mice

**Figure 5:**
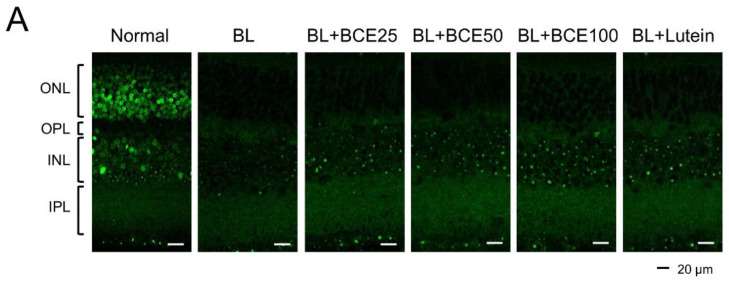

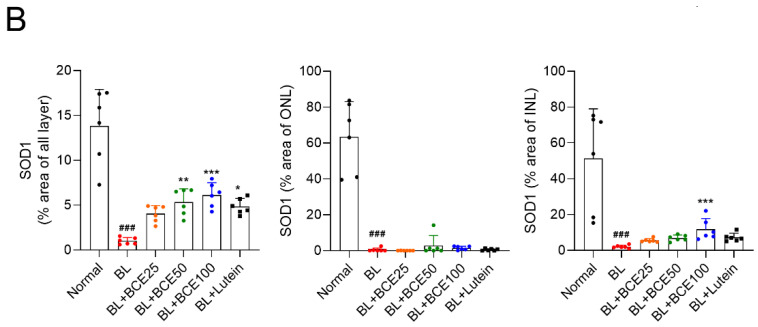
Effects of BCE on SOD1 expression in the BL-induced retinal degeneration mouse model. (**A**) Immunofluorescence staining to analyze SOD1 expression in all layers of the retina. Sections between 100 and 200 μm from the optic nerve of both eyes were observed. (**B**) The SOD1 protein levels were quantified using Image J. INL, inner nuclear layer; IPL, inner plexiform layer; ONL, outer nuclear layer; OPL, outer plexiform layer. The results are presented as the mean ± standard deviation. (*n* = 6). ^###^ *p* < 0.001 vs. normal group. * *p* < 0.05, ** *p* < 0.01, *** *p* < 0.001 vs. BL group.

To determine the effect of BCE on antioxidant activity in mice, SOD1 expression was analyzed by IHC (Figure 5A). A 2-week exposure to BL significantly (*p* < 0.001) reduced SOD1 expression level in the retina compared to that in the retina of the normal group. However, BCE administration in the BL-exposed group rescued SOD1 expression (*p* < 0.001) in a dose-dependent manner (Figure 5B). These data suggest that BCE has a positive effect on antioxidant activity.

## 4. Discussion

AMD is a visual disease that is considered as the third leading cause of visual impairment after cataracts and glaucoma, and its prevalence increases with age [21]. With the growing health awareness and concern of an aging society, interest in age-related diseases has increased. Moreover, with the advent of the smart era, we are constantly exposed to BL as we encounter numerous electronic devices in our daily lives. In this study, in vitro and in vivo BL-induced retinal damage models were used to investigate the protective effects of BCE on the retina. BCE treatment protected ARPE-19 cells from A2E and BL-induced phototoxicity. Additionally, oral administration of BCE ameliorated ocular abnormalities, suppressed retinal damage, and improved SOD1 expression in the retina after 2 weeks of BL exposure. These data demonstrated the retinal protective effects of BCE. Anthocyanins from members of the Ericaceae family are traditionally known for their antioxidant, anti-inflammatory, vasoprotective, and immune-activating properties [22,23,24,25]. Recently, their strong antioxidant properties have raised interest in their applicability in the treatment of phototoxic diseases of the eye caused by BL or UV radiation. Bilberry (*Vaccinium myrtillus* L) and lingonberry (*Vaccinium vitis-idaea*) extracts inhibited photoreceptor cell damage caused by BL in cell experiments [26,27]. In addition, the extract of *Vaccinium uliginosum* L. has been shown to protect retinal pigment epithelial cells and the retina by inhibiting the accumulation and photooxidation of A2E, a type of lipofuscin, both in vitro and in vivo [28,29]. BC has been reported to contain anthocyanins with antioxidant properties [30]. The effect of BC juice on chronic ethanol-induced oxidative stress was previously investigated [31]. Administration of BC juice showed antioxidant metabolic effects by regulating the expression of peroxisome proliferator-activated receptor α, AMP-activated protein kinase, tumor necrosis factor α, and NF-κB, as well as the activities of Cu, Zn-superoxide dismutase, catalase, and glutathione. In the human dermal fibroblast cell line (NHDF), BC extracts reduced the ROS level increased by UVB irradiation by regulating the Nrf2/HO-1 signaling pathway [32]. In RAW264.7 cells, BC extract treatment showed an anti-inflammatory effect by reducing the mRNA levels of IL1B and IL6, which were increased by LPS stimulation [33]. Although BC has several benefits, such as antioxidant and anti-inflammatory properties [34], its effects on the retina remain unclear. In this study, we demonstrated the efficacy of BC in both in vitro and in vivo models of retinal degeneration induced by BL.

To date, several in vitro damage models using RPE cells subjected to chemical or oxidative stress have been reported. H_2_O_2_ treatment increases IL-6, IL-8, and vascular endothelial growth factor expression and decreases complement factor H expression in ARPE-19 cells [35,36]. These results are similar to those of inflammatory response activation, complement system dysregulation, and increased angiogenesis-related factors that are characteristic of AMD. Amyloid beta also inhibits cell proliferation and activates apoptosis by inactivating RAGE/NF-κB signaling in ARPE-19 cells [37,38]. NaIO_3_ induces necrosis in ARPE-19 cells [39]. Recently, many studies have reported that BL irradiation increases oxidative stress and free radicals in the retina and causes apoptosis of photoreceptor cells, thereby damaging the retina [40,41]. Thus, the BL-induced retinal damage mouse model is a representative animal model for screening drugs that inhibit dry AMD. Our in vivo findings demonstrated that BCE can effectively protect against retinal damage caused by BL irradiation. This positive effect resulted from the antioxidant activity of anthocyanins, which are the main bioactive compounds in BCE. The major anthocyanins of BCE, D3R, D3G, C3R, and C3G, have been reported to have strong antioxidant activities, such as the suppression of intracellular ROS production [42,43,44,45]. Additionally, the retinal protective effect of anthocyanins, such as bilberry anthocyanins, ameliorated retinal damage in a visible light-induced retinal degeneration model in pigmented rabbits by increasing antioxidant defense mechanisms and suppressing retinal cell apoptosis [46]. There are several mechanisms for removing oxidative stress from the body, including the retina. SOD is a catalytic enzyme that removes superoxide radicals by alternately catalyzing the dismutation of the superoxide radical into ordinary molecular oxygen and hydrogen peroxide [47]. There are three isozymes in humans and mammals: SOD1, SOD2, and SOD3, of which SOD1 has the highest amount and activity in the retina [47]. As previously reported, mice deficient in SOD1 develop drusen formation and RPE dysfunction [48]. Our in vivo results showed that BCE upregulated SOD1 expression in all retinal layers and decreased the number of mice in which drusen was formed.

Anthocyanins in BCE are absorbed in their intact forms upon oral administration and distributed in ocular tissues, including the cornea, aqueous humor, iris, ciliary body, choroid, sclera, and retina [49]. After oral administration of blackcurrant extract, the anthocyanins D3R, D3G, C3R, and C3G were detected in the whole eye. These four types are the major anthocyanins present in the BCE used in this study. Therefore, BCE, because of its antioxidant activity and bioavailability that can be distributed to ocular tissues, has the potential to be used as a health supplement to protect against retinal damage from BL exposure. Lutein has long been considered as a dietary supplement to reduce the risk of AMD. However, BCE (>750 mg/mL in water) shows higher water solubility than lutein (insoluble in water) [50]. Therefore, BCE can be consumed in the form of fruit juice or concentrate, and it is easy to process; thus, it has advantages in food industry. In addition, BCE has long been known to be a good source of vitamin C.

## 5. Conclusions

In this study, we demonstrated that blackcurrant (BC, *Ribes nigrum* L.) extract (BCE) protected ARPE-19 cells in in vitro and in vivo models of retinal degeneration. BCE inhibited A2E accumulation in ARPE-19 cells and significantly downregulated the expression of genes upregulated by A2E and BL. In vivo, oral administration of BCE ameliorated ocular lesions of blue light (BL)-induced retinal damage and rescued the thickness of the whole retina and photoreceptor segment, outer nuclear, and inner nuclear layers. Additionally, BCE administration rescued BL-induced reduction in SOD1 expression levels. Taken together, our results suggest that the antioxidant activity of BCE and the inhibitory activity of lipofuscin accumulation in the retina might exert preventive and therapeutic effects on dry AMD.

## Figures and Tables

**Table 1:** Ophthalmological examination of mice.

		Groups					
Organs	Lesions	Normal	BL	BL + BCE25	BL + BCE50	BL + BCE100	BL + Lutein
Right eye	abnormalities observed	0/10	6/10	5/10	4/10	4/10	3/10
	retinal pigmentation	0/10	1/10	0/10	0/10	0/10	1/10
	retinal hyperemia	0/10	6/10	5/10	4/10	4/10	2/10
	drusen	0/10	3/10	2/10	2/10	1/10	1/10
Left eye	abnormalities observed	0/10	5/10	4/10	6/10	4/10	3/10
	retinal pigmentation	0/10	1/10	0/10	0/10	0/10	0/10
	retinal hyperemia	0/10	5/10	4/10	4/10	3/10	2/10
	drusen	0/10	3/10	2/10	2/10	1/10	1/10

Number of animals with lesions/number of animals examined. BL, blue light; BCE25, BCE 25 mg/kg; BCE50, BCE 50 mg/kg; BCE100, BCE 100 mg/kg; Lutein, lutein 4.6 mg/kg.

## Data Availability

The data are contained within the article and Supplementary Materials.

## Supplementary Materials

The following supporting information can be downloaded at: https://www.mdpi.com/article/10.3390/antiox11050832/s1, Figure S1: Relative mRNA levels for NF-κB signaling-related genes; Figure S2: Relative mRNA levels for inflammation- and inflammasome-related genes; Figure S3: Relative mRNA levels for cholesterol homeostasis-related genes; Figure S4: Relative mRNA levels for protein folding-related gene; Table S1: Composition of anthocyanins in BCE; Table S2: Primer sequences used for RT-qPCR.
