# Supplementary material for: Protective Effect of Ribes nigrum Extract against Blue Light-Induced Retinal Degeneration In Vitro and In Vivo
Source: Antioxidants (Basel). 2022 Apr 25;11(5):832. doi: 10.3390/antiox11050832 (PMC9137918; doi:10.3390/antiox11050832)
Supplement: Supplementary file 1 [file antioxidants-11-00832-s001.zip › antioxidants-1661120-supplementary.pdf]

Supplementary Information

Supplementary Figures

Figure S1

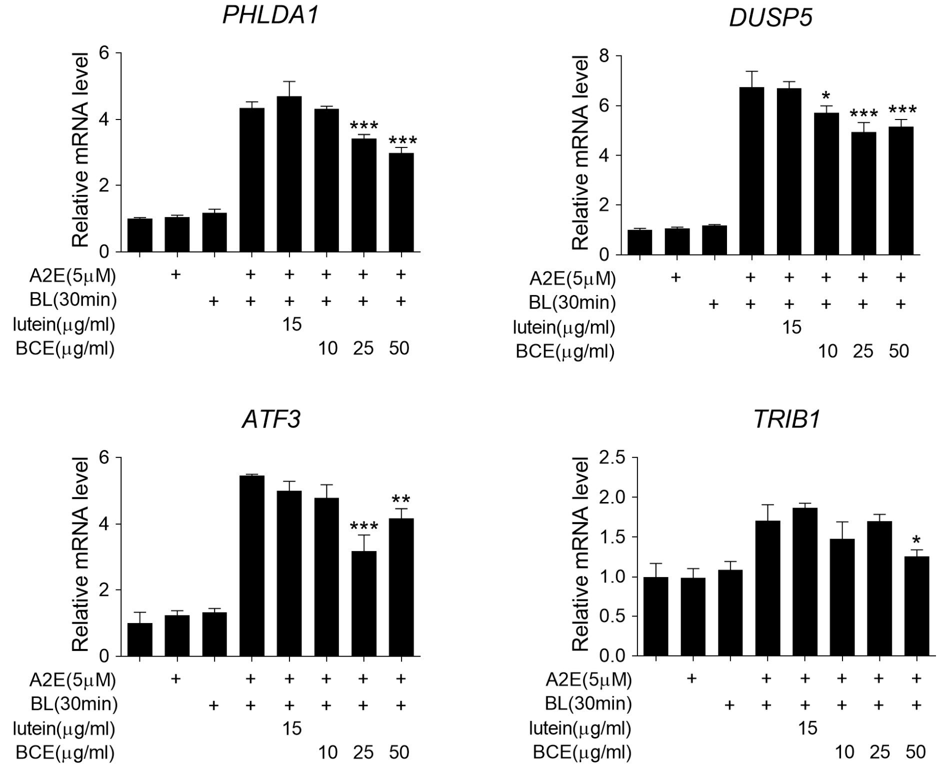

Supplementary Figure S1. Relative mRNA levels for NF-κB signaling-related genes. The results are presented as mean ± S.D. (n = 3). * $p < 0.05$, ** $p < 0.01$, *** $p < 0.001$ vs. A2E + BL.

Figure S2

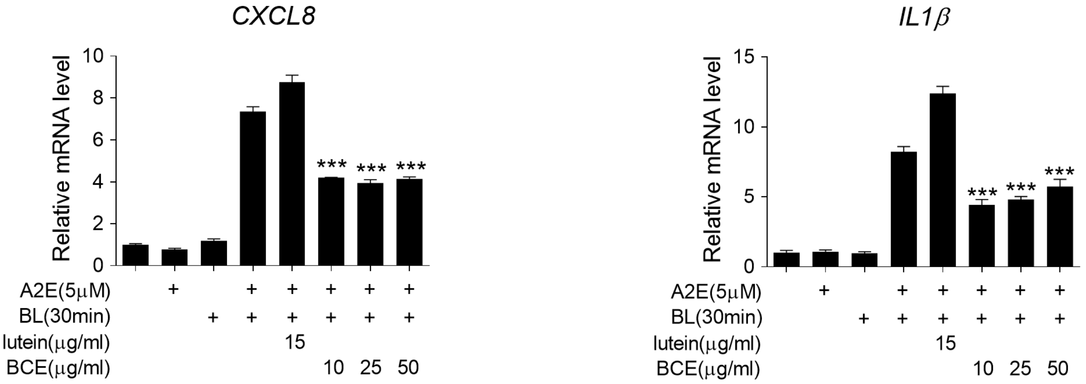

Supplementary Figure S2. Relative mRNA levels for inflammation and inflammasome-related gene. The results are presented as mean ± S.D. (n = 3). *** $p < 0.001$ vs. A2E + BL.

Figure S3

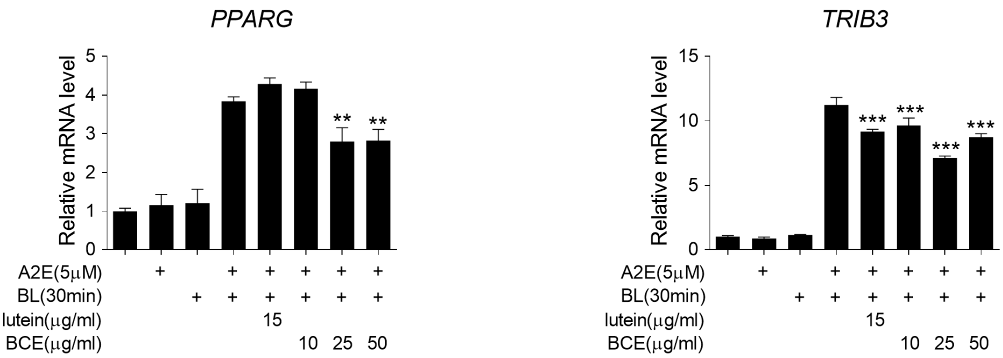

Supplementary Figure S3. Relative mRNA levels for cholesterol homeostasis-related genes. The results are presented as mean ± S.D. (n = 3). ** $p < 0.01$, *** $p < 0.001$ vs. A2E + BL.

Figure S4

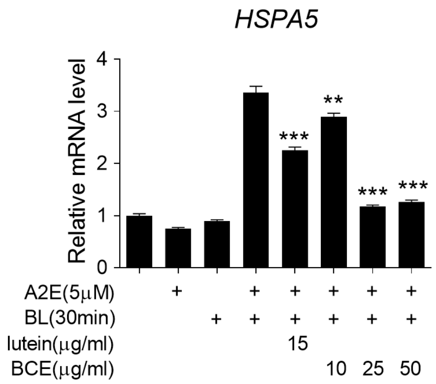

Supplementary Figure S4. Relative mRNA levels for protein folding-related gene. The results are presented as mean ± S.D. (n = 3). ** $p < 0.01$, *** $p < 0.001$ vs. A2E + BL.

Supplementary Tables

Supplementary Table S1. Composition of anthocyanins in BCE.

Anthocyanins	Contents (mg/g)
Total anthocyanins	391.26 ± 2.81
Delphinidin-3-rutinoside (D3R)	147.98 ± 2.81
Delphinidin-3-glucoside (D3G)	60.16 ± 1.06
Cyanidin-3-rutinoside (C3R)	146.77 ± 2.77
Cyanidin-3-glucoside (C3G)	35.07 ± 0.65

The results are presented as the mean ± standard deviation of three independent experiments (n = 3).

Supplementary Table S2. Primer sequences used for RT-qPCR.

Gene	Accession #	Forward	Reverse
PHLDA1	*NM_007350.3*	GGGCAAGACAAGGTTTTGAGGA	TCGCAAGTTTTCAGTAGGGTGA
DUSP5	*NM_004419.4*	GTCCTCACCTCGCTACTC	GGGCTCTCTCACTCTCAAT
ATF3	*NM_001674.4*	GCTGTCACCACGTGCAGTATCTCA	CTGTTCCTCCTCTTGCTGACAAGC
TRIB1	*NM_025195.4*	TTCAAGCAGATTGTCTCCGC	AGTGGTGTTGAGGATCTCAG
CXCL8	*NM_000584.4*	TGAATTACGGAATAATGAGTTAGAAC	TCAACCAGCAAGAAATTACTAAT
IL1B	*NM_000576.3*	CCACCTCCAGGGACAGGATA	AACACGCAGGACAGGTACAG
PPARG	*NM_138712.5*	GGGATCAGCTCCGTGGATCT	TGCACTTTGGTACTCTTGAGGTT
TRIB3	*NM_021158.5*	GAGGAGGGAGACAGAGAAG	TGGAAGGCACTGAAGGTT
HSPA5	*NM_005347.5*	AGCTGTAGCGTATGGTGCTG	AAGGGGACATACATCAAGCAGT
18S	*M10098.1*	GAGGATGAGGTGGAACGTGT	TCTTCAGTCGCTCCAGGTCT
